# Guard cell K^+^ channels of *Kalanchoë* follow the diel cycle of crassulacean acid metabolism

**DOI:** 10.1093/plphys/kiae506

**Published:** 2024-09-26

**Authors:** Cécile Lefoulon, Michael R Blatt

**Affiliations:** Laboratory of Plant Physiology and Biophysics, University of Glasgow, Bower Building, Glasgow G12 8QQ, UK; Laboratory of Plant Physiology and Biophysics, University of Glasgow, Bower Building, Glasgow G12 8QQ, UK

## Abstract

The activity of outward-rectifying but not inward-rectifying K + channels of *Kalanchoë* stomata follows the diel cycle of crassulacean acid metabolism.

Dear Editor,

Stomata are pores that open to allow gaseous diffusion between the atmosphere and the air space within the leaf and close to prevent excessive transpirational water loss (Jezek and Blatt 2017). In most angiosperm plants, CO_2_ entering through the pore diffuses to the mesophyll where it is fixed by RuBisCO. When open, the stomatal pore also allows for the diffusion of O_2_ and water vapor, both potentially problematic for the plant. Excessive water lost via transpiration through stomata risks leaf drying (Matthews and Lawson 2019; Blatt et al. 2022), while O_2_ competes with CO_2_ for fixation by Rubisco (Moroney et al. 2013).

Plant that use crassulacean acid metabolism (CAM) circumvent these risks by temporarily fixing CO_2_ in malic acid (Mal) and storing Mal in the vacuole at night; during the day, Mal is then released and decarboxylated to yield CO_2_ for fixation by the Rubisco (Borland et al. 2009; Hartwell et al. 2016). Many of the key metabolic enzymes that contribute to Mal synthesis, storage, and breakdown in the mesophyll are regulated by a diel cycle of gene transcription (Abraham et al. 2016; Boxall et al. 2017; Yang et al. 2017).

To accommodate this pattern of CO_2_ fixation, the stomata of CAM plants exhibit an inverted cycle, closing during the daytime to concentrate CO_2_ within the leaf and enhance carbon fixation by Rubisco while avoiding water loss by transpiration. They open during the night for CO_2_ entry, fixation, and temporary storage of Mal in the vacuole. Stomatal movements are driven in large part through ion transport by the guard cells surrounding the stomatal pore, opening and closing mediated by uptake and loss, respectively, of inorganic ions. We know that the anion channels of CAM guard cells are modulated through a diel cycle of gene transcription and translation complementary to the enzymes of Mal metabolism in the mesophyll. However, this pattern of gene expression does not extend to the predominant K^+^ channels; these channels do not exhibit substantial diel changes in transcript abundance (Abraham et al. 2016; Yang et al. 2017; Boxall et al. 2020).

We examined the K^+^ channels of guard cells in the model CAM species *Kalanchoë fedtschenkoi* and *Kalanchoë laxiflora* by two-electrode voltage clamp after impaling guard cells isolated in epidermal peels (Blatt 1987; Lefoulon et al. 2020). The characteristics of the *Kalanchoë* K^+^ channels proved to be similar to those of plants that rely on C_3_ and C_4_ photosynthesis detailed in the past, including *Arabidopsis thaliana, Vicia faba, Zea mays, Gynandropsis gynandra*, and *Nicotiana tabacum* (Blatt 1990; Fairley-Grenot and Assmann 1992; Roelfsema and Prins 1997; Blatt et al. 1999; Silva-Alvim et al. 2023). Notably, the outward-rectifying K^+^ current (Fig. 1) activated at voltages positive of the prevailing K^+^ equilibrium voltage, E_K_, and shifted, positive-going, with increasing external K^+^ concentration; the inward-rectifying K^+^ channels activated at voltages negative of −120 mV, a voltage-dependence that was independent of the K^+^ concentration outside, and both currents were blocked by 20 mm tetraethylammonium chloride (Supplementary Fig. S1).

**Figure 1.**
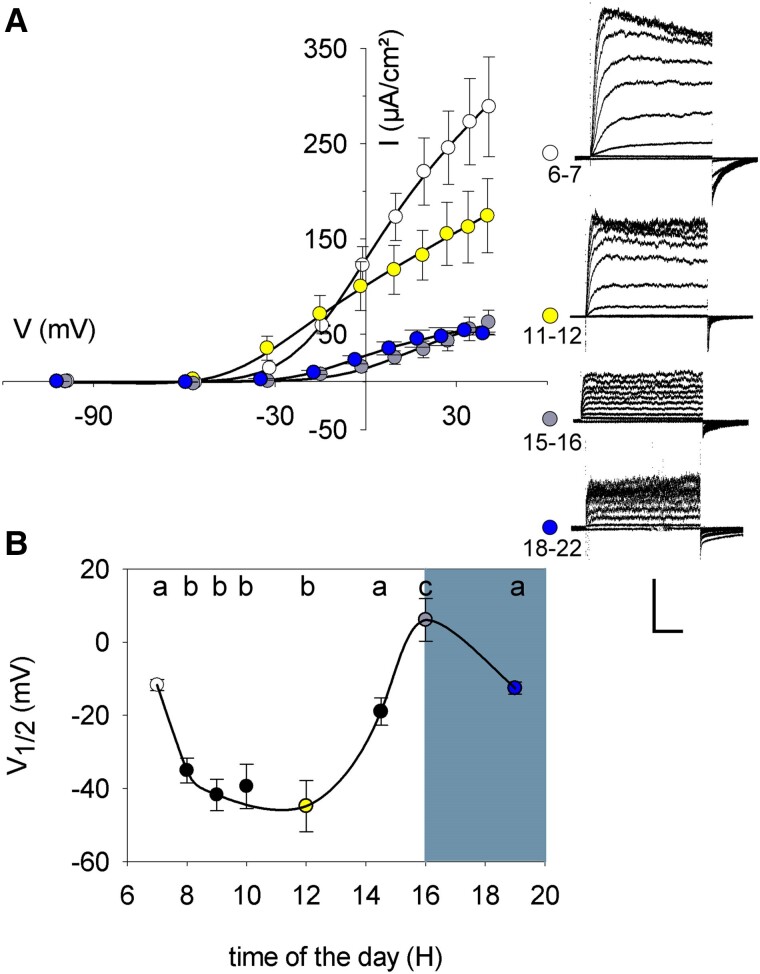
Outward-rectifying K^+^ currents of *K. fedtschenkoi* vary with the diel cycle. **A)** Mean steady-state current-voltage (I/V) curves (*left*) and representative clamp current traces (*right*) recorded at times between 6 and 22 h over the diel cycle. Plants were grown and measurements were carried out as described previously (Lefoulon et al. 2020) by 2-electrode voltage clamp of guard cells in epidermal peels superfused with 5 mm Ca^2+^-MES (2-(N-morpholino)propanesulfonic acid), pH 6.1 (1 mm Ca^2+^) and 10 mm KCl. Microelectrodes were filled with 200 mm K^+^ acetate, pH 7.5 (Blatt 1987). Currents were analyzed using Henry IV EP suite and corrected for guard cell surface area. Data points are color-coded, each point the mean ± SE of *n* > 7 for each data point, each from guard cells on different plants. Scale (*traces*): 100 µA vertical, 1 s horizontal. Curves are fittings of the mean currents to the Boltzmann function[1]$I=G_{\max}(V\text{-}E_{K})/(1+\exp(\delta F(V\text{-}V_{1/2})/RT))\;[1]$ where *G*_max_ is the maximum conductance, *V*_1/2_ is the midpoint voltage where *G* = 0.5 G_max_, *δ* is the apparent gating charge and defines voltage sensitivity around *V*_1/2_, *F* is the Faraday constant, *R* is the gas constant, and *T* is the temperature in K. The value for *δ* was 2.4 ± 0.4 and was held in common for fitting. **B)** Means ± SE of values for *V*_1/2_ from the fittings in (A) plotted as a function of diel time. Shading marks the dark period of the diel cycle. Lettering (*above*) indicates significant differences at *P* < 0.001 by post hoc Tukey analysis.

We examined the steady-state currents collected at different time points in the diel cycle and fitted these to a Boltzmann function (Fig. 1). For the outward-rectifying channels, the analysis showed that the voltage sensitivity, measured as V_1/2_, followed a distinct cycle, beginning early in the daylight with values near −10 mV in 10 mm K^+^, similar to the characteristics from non-CAM plants (Blatt and Gradmann 1997; Papanatsiou et al. 2016; Horaruang et al. 2022); later in the daylight period, V_1/2_ values shifted to voltages near −40 to −50 mV before rising to a mean of +6 ± 5 mV around 16 h and returning to a mean near −10 mV around 19 h. The analysis also showed a similar variation in the maximum ensemble conductance, *G*_max_, the greatest *G*_max_ for the outward-rectifying current evident in the first 6 h of daylight and declining thereafter to a minimum during the nighttime (Supplementary Fig. S2). By contrast, the inward-rectifying K^+^ current showed no significant changes in either V_1/2_ (Supplementary Fig. S3) or *G*_max_ over the diel cycle. These results indicate that the outward K^+^ current regulates to favor K^+^ efflux during the day and suppress the same activity at night: displacing V_1/2_ to more negative voltages effectively reduces the energy barrier for channel activation, thereby enhancing the capacity for K^+^ efflux in the daytime; conversely, reducing *G*_max_ during the dark period limits the overall capacity for efflux, so biasing the membrane for solute uptake to open the stomata. Of course, the actual flux of K^+^ will also be governed by the prevailing membrane voltage and, hence, by charge movements through other membrane transporters. At present, there are no data for membrane voltage in any CAM plant and, hence, we can only speculate on the likely K^+^ flux at any given time. Even so, our results show that the *capacity* for flux through the outward-rectifying K^+^ channels—indicated by the steady-state current-voltage curves recorded with voltage brought under control by the voltage clamp—is strongly modulated over the diel cycle.

Boxall et al. (2020) developed RNAi lines of *K. laxiflora* impaired in CAM metabolism that yielded important insights into the diel patterns of anion channel regulation (Lefoulon et al. 2020). We used 2 phosphoenol pyruvate (PEPC) mutant lines that suppress CO_2_ fixation, the RNAi knockdown rPEPC-A mutant, and the rPEPC-B null mutant line. Both lines were shown to alter the diel regulation of stomatal aperture, rPEPC-A impairing closing and opening during the second half of the daylight period, and rPEPC-B yielding a complete loss of the inverted CAM stomatal cycle (Boxall et al. 2020; Lefoulon et al. 2020).

Although we could not resolve a significant change in V_1/2_, we found that the *G*_max_ for the outward-rectifying K^+^ current in *K. laxiflora* guard cells, as in *K. fedtschenkoi*, was enhanced during the daylight period, typically by 3.5-fold, compared to the dark period of the cycle (Fig. 2). These results contrast with the transcript profile of outward-rectifiers potassium channels GORK and SKOR of *K. laxiflora*, the former varying by roughly 50% over the diel cycle and the latter declining barely 2-fold at night compared to the daytime (Boxall et al. 2020). In the rPEPC-A mutant, by contrast, we observed no significant differences in *G*_max_, and in the rPEPC-B mutant, the *G*_max_ cycle was reversed when compared to the wild-type plants (Fig. 2). We also noted that the inward-rectifying K^+^ current was reduced in amplitude in both rPEPC mutants when compared with wild-type *K. laxiflora* guard cells. So, the analysis suggests that both K^+^ channels are linked to Mal metabolism, with the outward-rectifying K^+^ channels closely tied to the cycle of stomatal opening and closing. In summary, our studies show a clear variation in ensemble channel activities, especially of the outward-rectifying K^+^ channels, that are consistent with the expected changes in K^+^ flux capacity over the CAM diel cycle. Furthermore, we can show that mutants suppressed in Mal metabolism also partially or fully suppress these diel changes in K^+^ channel capacity. While the findings complement our previous analysis of the CAM guard cell anion current, they also underscore important differences. Notably, whereas the *Kalanchoë* SLAC1 and R-type anion channels are closely tied to a diel cycle of transcriptional control and disconnected from mesophyll photosynthesis (Lefoulon et al. 2020), the K^+^ channels appear to be regulated by other mechanisms that are sensitive to the CAM cycle of Mal metabolism.

**Figure 2.**
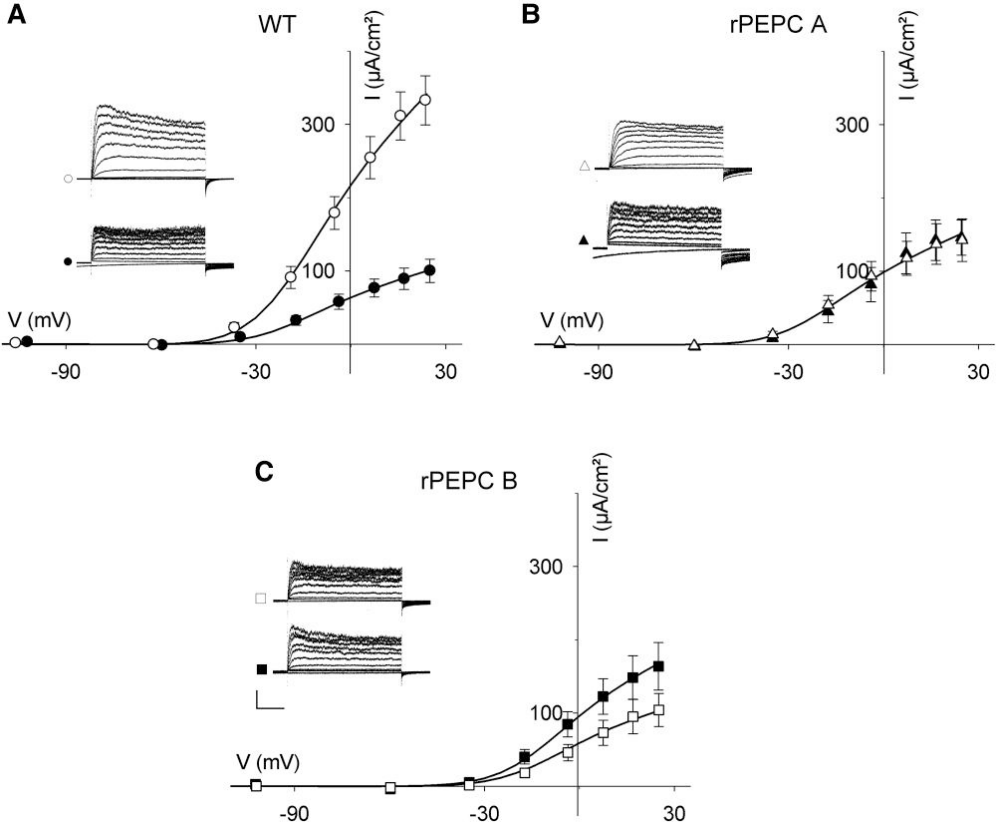
The diel cycle in outward-rectifying K^+^ current of *K. laxiflora* is suppressed or reversed in rPEPC mutants that affect mesophyll Mal metabolism. Mean steady-state current-voltage (I/V) curves recorded as in Fig. 1. Scale (*traces*): 100 µA vertical, 1 s horizontal. Current means ± SE (*n* > 8) shown were recorded at 6 h (open symbols) and 20 h (filled symbols) of the diel cycle, marking the light–dark maxima–minima only shown for clarity. Data are for the wild-type (A), and the mutants rPEPC-A (B) and rPEPC-B (C). Data are means ± SE of *n* > 8 each from guard cells on different plants for each genotype. Curves are fittings to Eqn. [1] with *δ* held in common (*δ* = 2.4 ± 0.2) and *V*_1/2_ values between night and day for each genotype. Values for *V*_1/2_ are −21 ± 0.9 mV (wt), −24 ± 1.7 mV (rPEPC-A), and −14 ± 1.4 mV (rPEPC-B).

## Supplementary Material

kiae506_Supplementary_Data

## Data Availability

All data are available from the authors on reasonable request.

## Supplementary data

The following materials are available in the online version of this article.

**Supplementary Figure S1.** Outward- and inward-rectifying currents of *K. fedtschenkoi* are inhibited by the K^+^ channel blocker tetraethylammonium chloride and the outward-rectifying current shows K^+^-dependent gating.

**Supplementary Figure S2.** Diel variation in *G*_max_.

**Supplementary Figure S3.** Inward-rectifying K^+^ currents of *K. fedtschenkoi* vary little with the diel cycle.

## Dive Curated Terms

The following phenotypic, genotypic, and functional terms are of significance to the work described in this paper:

GORK Gramene: AT5G37500
GORK Araport: AT5G37500

SKOR Gramene: AT3G02850
SKOR Araport: AT3G02850

PEPC Gramene: Sobic.010G160700
PEPC Araport: Sobic.010G160700

SLAC1 Gramene: AT1G12480
SLAC1 Araport: AT1G12480

SKOR related in Kalanchoe laxiflora Gramene: Kalax.0729s0016
SKOR related in Kalanchoe laxiflora Araport: Kalax.0729s0016

SKOR related in Kalanchoe laxiflora 2 Gramene: Kalax.0137s0050
SKOR related in Kalanchoe laxiflora 2 Araport: Kalax.0137s0050

GORK related in Kalanchoe laxiflora Gramene: Kalax.0408s0032
GORK related in Kalanchoe laxiflora Araport: Kalax.0408s0032

GORK related in Kalanchoe laxiflora 2 Gramene: Kalax.0157s0029
GORK related in Kalanchoe laxiflora 2 Araport: Kalax.0157s0029

GORK related in Kalanchoe fedtschenkoi Gramene: Kaladp0278s0021
GORK related in Kalanchoe fedtschenkoi Araport: Kaladp0278s0021

SKOR related in Kalanchoe fedtschenkoi Gramene: Kaladp0087s0150
SKOR related in Kalanchoe fedtschenkoi Araport: Kaladp0087s0150
