# Supplementary material for: Guard cell K+ channels of Kalanchoë follow the diel cycle of crassulacean acid metabolism
Source: Plant Physiol. 2024 Sep 26;196(4):2300–3. doi: 10.1093/plphys/kiae506 (PMC11638099; doi:10.1093/plphys/kiae506)
Supplement: kiae506_Supplementary_Data [file kiae506_supplementary_data.pdf]

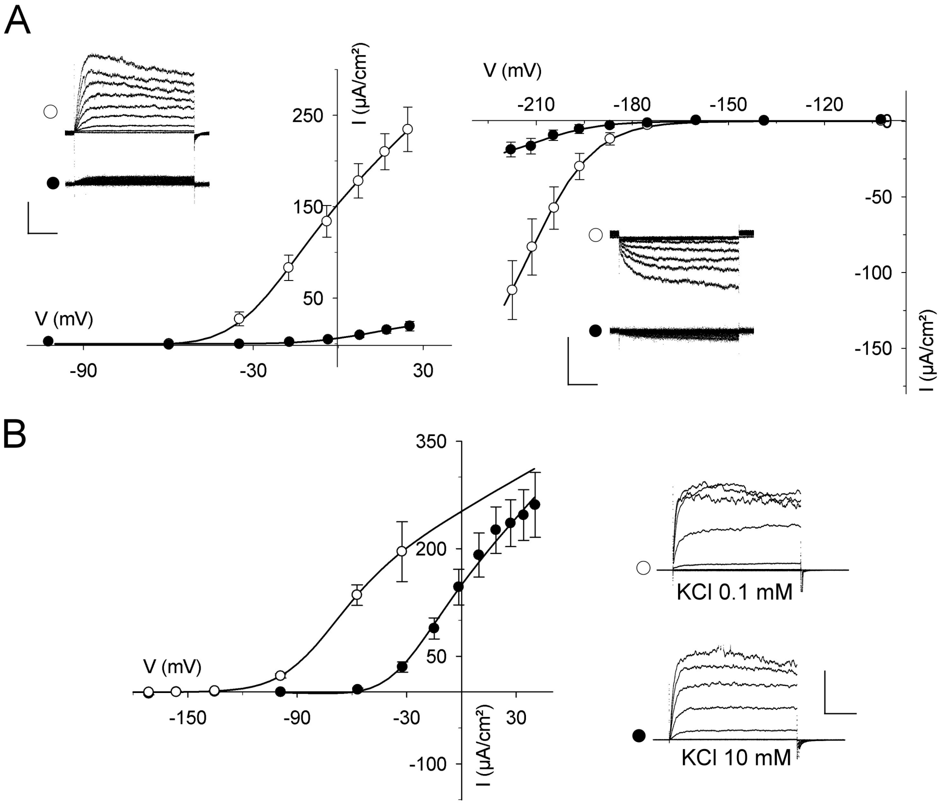

Supplemental Figure S1. Outward- and inward-rectifying currents of *Kalanchoë fedtschenkoi* are inhibited by the K^+ channel blocker tetraethylammonium chloride and the outward-rectifying current shows K^+-dependent gating.

(A) Steady-state current-voltage (I/V) curves for the inward- and outward-rectifying currents in 10 mM K^+ before (open symbols) and after (filled symbols) adding 20 mM tetraethylammonium chloride to the bath. Representative current traces are shown as insets. Scale: 100 µA/cm² vertical, 1 s horizontal.

(B) Mean steady-state current-voltage curves and representative traces (*insets*) recorded in guard cells bathed in 0.1 mM (open circles) and then in 10 mM K^+ (filled circles). Scale: 100 µA/cm² vertical, 1 s horizontal. Current-voltage data are means ±SE of n>4 each from guard cells on different plant. Curves are fittings to the Boltzmann function of Eqn [1] yielding $V_{1/2}$ of -78±4 mV in 0.1 mM K^+, and -28±3 mV in 10 mM K^+.

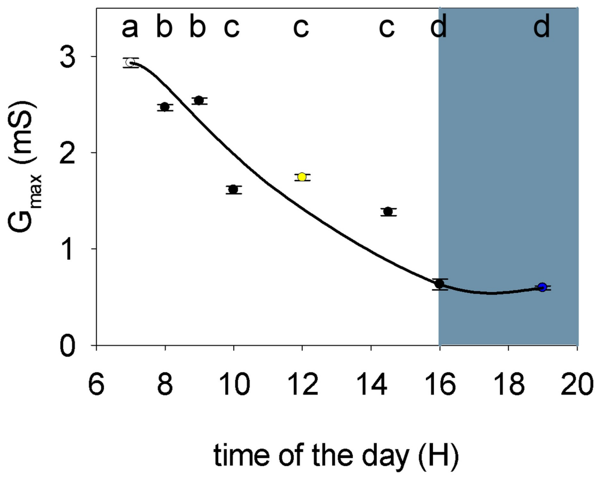

Supplemental Figure S2. Diel variation in G_{max}.
Data are means ±SE of values from fittings in Fig. 1. Shading indicates nighttime. Letters indicate significant differences at P<0.001. The curve shown is for visual guidance only.

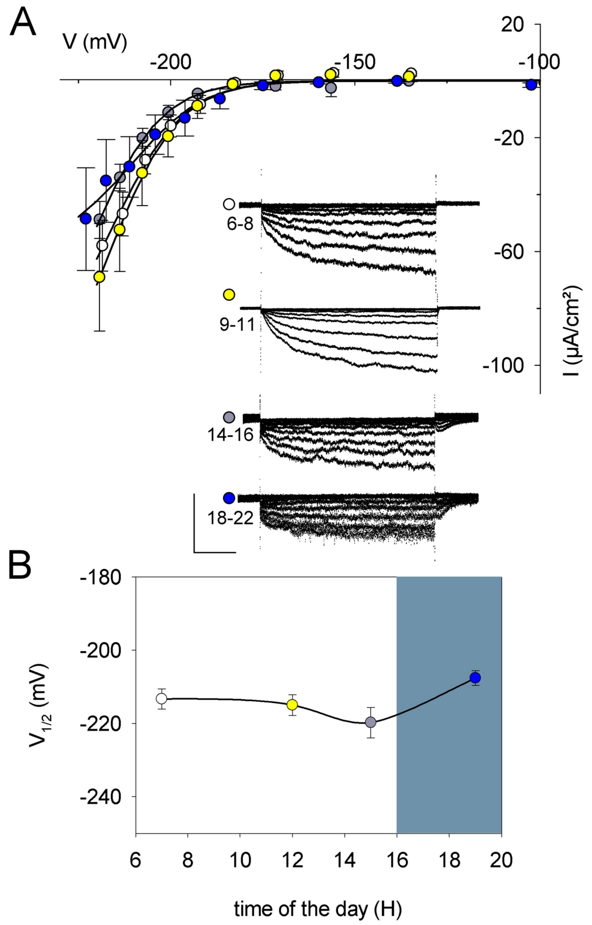

Supplemental Figure S3. Inward-rectifying K^+ currents of *Kalanchoë fedtschenkoi* vary little with the diurnal cycle.

(A) Mean steady-state current-voltage (I/V) curves (*left*) and representative clamp current traces (*right*) recorded at times between 6 and 22 h over the diurnal cycle. Data collected and analysed together with those of Figure 1 and are mean ±SE of n>7 each from guard cells on different plant. Scale (*traces*): 100 µA vertical, 1 s horizontal. Curves are fittings of the mean currents to the Boltzmann function of Eqn [1].

(B) Means ±SE of values for $V_{1/2}$ from the fittings in (A) plotted as a function of diurnal time. Shading marks the dark period of the diurnal cycle. They are not significatively different (P = 0.085 ANOVA)
